# Immunopeptidomic Profiling of HLA‐A2‐Positive Triple Negative Breast Cancer Identifies Potential Immunotherapy Target Antigens

**DOI:** 10.1002/pmic.201700465

**Published:** 2018-06-27

**Authors:** Nicola Ternette, Marloes J. M. Olde Nordkamp, Julius Müller, Amanda P. Anderson, Annalisa Nicastri, Adrian V. S. Hill, Benedikt M. Kessler, Demin Li

**Affiliations:** ^1^ The Jenner Institute University of Oxford Oxford OX3 7FZ UK; ^2^ Target Discovery Institute Nuffield Department of Medicine Oxford OX3 7FZ UK; ^3^ Nuffield Division of Clinical Laboratory Sciences Radcliffe Department of Medicine University of Oxford Oxford OX3 9DU UK

**Keywords:** aTaCC, breast cancer, HLA‐A2, immunopeptidome, mass spectrometry

## Abstract

The recent development in immune checkpoint inhibitors and chimeric antigen receptor (CAR) T‐cells in the treatment of cancer has not only demonstrated the potency of utilizing T‐cell reactivity for cancer therapy, but has also highlighted the need for developing new approaches to discover targets suitable for such novel therapeutics. Here we analyzed the immunopeptidomes of six HLA‐A2‐positive triple negative breast cancer (TNBC) samples by nano‐ultra performance liquid chromatography tandem mass spectrometry (nUPLC‐MS^2^). Immunopeptidomic profiling identified a total of 19 675 peptides from tumor and adjacent normal tissue and 130 of the peptides were found to have higher abundance in tumor than in normal tissues. To determine potential therapeutic target proteins, we calculated the average tumor‐associated cohort coverage (aTaCC) that represents the percentage coverage of each protein in this cohort by peptides that had higher tumoral abundance. Cofilin‐1 (CFL‐1), interleukin‐32 (IL‐32), proliferating cell nuclear antigen (PCNA), syntenin‐1 (SDCBP), and ribophorin‐2 (RPN‐2) were found to have the highest aTaCC scores. We propose that these antigens could be evaluated further for their potential as targets in breast cancer immunotherapy and the small cohort immunopeptidomics analysis technique could be used in a wide spectrum of target discovery. Data are available via ProteomeXchange with identifier PXD009738.

## 1 Introduction

Triple negative breast cancers (TNBC), defined by the tumor's lack of expression of estrogen receptors (ER), progesterone receptors (PR), and human epithelial receptor‐2 (HER2), represent an aggressive subtype of breast cancer.^1^ TNBC comprise 10–20% of breast cancer cases, and patients have poorer survival rates compared with other subtypes.^2^ Due to their hormone independency, the treatment of TNBC is currently limited to cytotoxic therapy.^3^ As such, there is an urgent need to develop novel therapies for this type of breast cancer and recent developments in immunotherapy provide the potential for much needed new opportunities.

Immunotherapy relies on the immune cells’ recognition of protein and/or peptide targets presented on cancer cells. The peptides presented by major histocompatibility complex (MHC) class I, which are routinely surveyed by T lymphocytes, represent an attractive class of immunotherapy targets. The immunopeptidome, the collection of the peptides presented by human leucocyte antigens (HLA), the human MHC, comprises thousands of peptides derived from degradation of cellular proteins, and is a reflection of the protein expression repertoire of the cells.^4^, ^5^ Cancer cells, due to their aberrant expression of proteins that enable them to outgrow the normal tissue and to survive immune surveillance, are suggested to present a different set of peptides in comparison to normal tissues. Epitopes derived from tumor‐associated antigens (TAAs), which have low or minimal expression on normal cells, and tumor‐specific antigens (TSAs), which are only expressed by malignant cells, are effectively presented by cancer cells and efficiently induce specific T‐cell activation.^5^, ^6^ Such peptides are the foundation for T‐cell based immunotherapy, including cancer vaccines, chimeric antigen receptor (CAR) T‐cells, soluble T‐cell receptors (TCRs), and T‐cell receptor mimic (TCRm) antibodies.^7^

Immunopeptidome studies carried out using model breast cancer cell lines have yielded a significant number of peptides.^8^ By comparing immunopeptidomes from breast cancer cell lines and a nontumorigenic immortalized cell line, Hawkins et al. identified a number of cancer‐specific epitopes including a peptide derived from macrophage migration inhibitory factor (MIF),^9^ for which a TCRm antibody was subsequently developed that was shown to reduce tumor burden both in vitro and in vivo.^10^ Although such cell line studies have generated a significant amount of information,^11^ primary tissues are generally more desirable as they have not gone through the extended culture and propagation under artificial conditions which inevitably affects the composition of the peptidomes. Early work on renal cell carcinoma provided a glimpse of a primary cancer immunopeptidome,^12^ and with the progress of mass spectrometry (MS) technology, more recent work on hematological malignancies, including acute myeloid leukemia,^13^ multiple myeloma,^14^ and chronic lymphocytic leukemia^15^ has generally detected tens of thousands of peptides and provided systematic views of cancer immunopeptidomes compared with matching normal tissues.

**Significance Statement**

Triple negative breast cancer is an aggressive subtype of breast cancer that urgently requires novel therapies. In this study, we performed immunopeptidomic profiling of patient tumor and adjacent normal tissues with the objective of identifying potential targets for immunotherapy development, which we believe is the first of such studies. High quality peptide data were obtained by nano‐ultra performance liquid chromatography tandem mass spectrometry (nUPLC‐MS^2^) from both tumor and adjacent normal tissues and a number of peptides were found to be common to all cancer tissues but absent in normal tissues. Based on peptide abundance data, we were able to calculate the presentation coverage of proteins by peptides preferentially found in tumor tissues. This approach prioritized five proteins, including cofilin‐1 (CFL‐1), interleukin‐32 (IL‐32), proliferating cell nuclear antigen (PCNA), syntenin‐1 (SDCBP), and ribophorin‐2 (RPN‐2), as potential therapeutic targets. The common tumor peptides and proteins identified in this study provide candidates for further validation which could provide targets for cancer vaccines, soluble T‐cell receptors, and T‐cell receptor mimic antibodies. Additionally this study also provides a novel approach for immunotherapy target discovery.

Here, we report our work on TNBC primary samples and matched adjacent normal breast tissues using advanced MS technology. We have identified a large number of peptides associated with HLA class I molecules in both cancer samples and normal tissues, and by comparing cancer tissues against the matched normal breast samples, we have identified a number of protein antigens that are more abundantly presented by cancer cells and have prioritized such antigens for further validation as potential targets for future immunotherapy development.

## 2 Experimental Section

### 2.1 Tissue Samples

TNBC samples and matched adjacent normal tissue samples were provided by Breast Cancer Now Tissue Bank, UK, who obtained patients’ consent for using these samples for this study (not including HLA genotyping). Sample‐related procedures were approved by Oxford University Research Ethics Committee (Reference number 04/Q1604/21). Electronic images of hematoxylin and eosin (H&E) stained TNBC biopsies provided by the Tissue Bank were examined and 15 cases were selected where a) the H&E staining showed a high proportion of tumor cells in the section (>40% of the section), b) the tissue morphology was maintained, and c) there was sufficient matched adjacent normal tissue available for further testing by mass spectrometry (Table 1). Frozen slides from the samples were requested for HLA‐A2 expression screening by immunohistochemistry using an HLA‐A2‐specific monoclonal antibody. Six pairs of TNBC and matching normal tissues were requested for mass spectrometry analysis. Samples were stored at −80 °C until use.

**Table 1:** Patient characteristics

Patient ID	Age	Sex	Diagnosis	HLA‐A2 staining	Tumor HLA‐A2 downregulation
1	53	F	Ductal NST	+	No
2	70	F	Pure special type—Basal	+	No
3	50	F	Ductal/NST	+	No
4	65	F	Ductal/NST	+	No
5	31	F	Ductal/NST	+	No
6	50	F	Ductal/NST	+	No
7	88	F	Ductal/NST	−	N/A
8	71	F	Ductal/NST	+	Yes
9	76	F	Ductal/NST	+	Yes
10	38	F	Ductal/NST	−	N/A
11	46	F	Ductal/NST	−	N/A
12	61	F	Classic basal with squamous areas	−	N/A
13	88	F	Ductal/metaplastic mixed	−	N/A
14	56	F	Ductal/NST	+	Yes
15	60	F	Ductal/NST	−	N/A

Abbreviations: NST, no‐specific type; N/A, not applicable.

### 2.2 HLA‐Associated Peptide Purification from Biopsy Material

Approximately 0.5 cm^3^ of breast cancer and matching adjacent normal tissue biopsy material was homogenized in lysis buffer (1% Igepal, 300 mM sodium chloride, 100 mM Tris, pH 8.0) supplemented with protease inhibitor cocktail (Roche) using a bead beater (Precellys 24 bead‐beater, Bertin Technologies) five times for 10 s at 6500 rpm. Lysates were cleared by subsequent centrifugation steps at 300 × *g* for 10 min and then 20 000 × *g* for 60 min. One milligram per sample of human anti‐HLA class I antibody (W6/32, ATCC HB‐95) was bound and cross‐linked to 1 mL Protein A beads (GE Healthcare) and used for immunoprecipitation of HLA complexes as described previously.^16^ In brief, lysates were incubated with the antibody beads overnight at 4 °C and washed subsequently with 50 mM Tris, pH 8.0 containing first 150 mM, then 450 mM and finally 0 mM NaCl. Peptides were eluted with 5 mL of 10% acetic acid. Dried peptides were resuspended and injected onto a 4.6 × 50 mm ProSwift RP‐1S column (Thermo Fisher Scientific). Peptides were separated from larger complex components by elution using a 500 μL min^−1^ flow rate over 10 min from 2 to 25% acetonitrile in 0.1% trifluoroacetic acid. Alternate fractions were pooled and two final fractions were analyzed by nano‐ultra performance liquid chromatography tandem mass spectrometry (nUPLC‐MS^2^).

### 2.3 Nano‐Ultra Performance Liquid Chromatography Tandem Mass Spectrometry (nUPLC‐MS^2^)

HLA‐peptides eluted from tissues were separated on an Ultimate 3000 RSLCnano system (Thermo Scientific) using a PepMap C18 column, 2 μm particle size, 75 μm x 50 cm (Thermo Scientific) with a 30 min (two technical replicates) and 1 h (single run) linear gradient of 3–25% buffer B (0.1% formic acid, 5% DMSO in acetonitrile) in buffer A (0.1% formic acid, 5% DMSO in water) at a flow rate of 250 μL min^−1^. Peptides were introduced using an EASY‐Spray source at 2000 V and to a Fusion Lumos (Thermo Scientific). The ion transfer tube temperature was set to 305 °C. Full MS spectra were recorded from 300 to 1500 *m*/*z* in the Orbitrap at 120 000 resolution with an automatic gain control (AGC) target of 400 000. Precursor selection was performed using TopSpeed mode at a cycle time of 2 s. Peptide ions were isolated using an isolation width of 1.2 amu and trapped at a maximal injection time of 120 ms with an AGC target of 300 000. Higher‐energy collisional dissociation (HCD) fragmentation was induced at an energy setting of 28 for peptides with a charge state of 2–4, while singly charged peptides were fragmented at an energy setting of 32 at lower priority. Fragments were analyzed in the Orbitrap at 30 000 resolution.

Each sample was analyzed in a 1 h gradient discovery run and two additional 30 min technical duplicates for quantitative analysis.

### 2.4 Data Analysis

Analysis of raw data was performed using Peaks 8.0 software (Bioinformatics Solutions). Sequence interpretation of MS^2^ spectra was carried out using databases containing all human Swiss‐Prot database entries (03/03/2016, 20210 entries). Peaks PTM searches were performed with all 485 build‐in modifications as defined by Peaks 8.0. Peptides with a length of less than seven amino acids were excluded from the analysis results and peptides with a Peaks score of below 15 were ignored. The false discovery rate (FDR) was estimated with randomized decoy database searches and ranged between 1.0 and 2.1% for all samples, with an average FDR of 1.4%. For quantitative analysis of peptides, normalized peak areas from all detected charge states and both analyzed HPLC fractions were added up if multiple values were measured to retrieve a final peptide peak area. For volcano plots, the *p‐*value from the regarding quantitative value with maximal area abundance was selected. Motif analysis of common amino acids in peptide sequences was performed using WebLogo 3.5 (weblogo.threeplusone.com). Peptide clustering was performed with GibbsCluster‐2.0,^17^ and peptide binding predictions were performed using NetMHCpan 4.0.^18^ The mass spectrometry proteomics data have been deposited to the ProteomeXchange Consortium via the PRIDE.^19^ partner repository with the dataset identifier PXD009738 and 10.6019/PXD009738.

## 3 Results

### 3.1 Immunopeptidome Profiling of TNBC and Adjacent Normal Tissue Samples

HLA‐A*0201 is one of the commonest HLA class I alleles in major human ethnic groups and we set out to investigate the HLA‐A*0201‐presented immunopeptidome in primary TNBC samples. Fifteen TNBC patients were pre‐screened for HLA‐A2 expression by immunohistochemistry using the anti‐HLA‐A2 antibody BB7.2 on frozen sections (Table 1). Nine out of the 15 samples were found to be HLA‐A2 positive, but 3 of the HLA‐A2‐positive samples (Patients 8, 9, and 14) showed significant tumoral HLA‐A2 downregulation (Figure S1, Supporting Information). HLA class I downregulation is a common mechanism for tumors to escape immune surveillance^20^ and it is reported to be correlated with worsening prognosis in breast cancer.^21^ These three samples were excluded from this study to enable effective immunoprecipitation. Finally, six pairs of tumor and adjacent normal tissue samples from HLA‐A2^+^ patients whose tumor samples did not show HLA‐A2 downregulation were collected. Samples were lysed and immunoprecipitated with a pan‐HLA class I antibody, and HLA‐associated peptides were eluted and analyzed by nUPLC‐MS^2^. The tumor‐adjacent normal tissue sample from Patient 6 failed immunoprecipitation due to technical reasons and therefore five paired tumor and normal tissues and one additional tumor sample were eventually analyzed.

**Figure 1:**
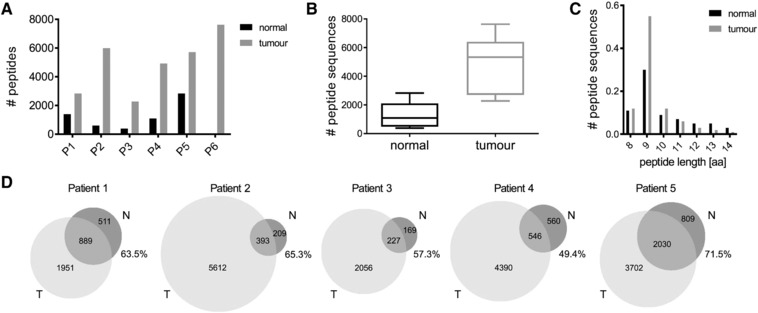
Characteristics of peptides isolated from HLA‐A2‐positive patient tumor and adjacent normal tissues. A) Numbers of HLA‐associated peptides identified from each patient sample. B) The average numbers of peptides identified from normal and tumor tissues from all patients. C) Length distribution of all identified peptides in normal and tumor tissue. D) Venn diagrams show the numbers of peptides identified in normal and tumor tissue for each patient. Percentage of peptides derived from normal tissue that are also present in the tumor sample of the same patient is shown for each graph.

Between 396 and 7635 peptide sequences were identified in each of the analyzed samples (Figure 1A, Table S1, Supporting Information), and overall, 19 675 distinct peptide sequences from 6275 distinct proteins were identified across all patients. Generally, more HLA‐peptides were identified in similar size breast cancer biopsies versus the adjacent normal tissue biopsies (*p* = 0.01; Figure 1B), which is likely due to the fact that the tumor tissues exhibit much higher numbers of HLA class I‐expressing cells in comparison to the fatty and fibrous connective tissue that forms normal breast tissue. Furthermore, the number of sequences with a length of 8–14 amino acids, the expected length of the majority of HLA class I‐associated peptides, represented 62–78% (average of 70%) and 87–96% (average of 91%) of the total peptide numbers for normal and tumor tissues, respectively (Figure 1C and Figure S2A, Supporting Information). Indeed, we observed relatively higher ratios of longer peptide sequences in the normal tissue samples for all patients (Figure S2A, Supporting Information and data not show for peptides >15 aa). These longer peptides can be degradation products of proteins that coprecipitate nonspecifically, which would explain the higher representation in normal tissue samples in which HLA‐bound peptide yield was low. Furthermore, longer peptides could originate from coprecipitating HLA class II molecules.^22^ The general charge state distribution of peptides as detected in the mass spectrometer was mostly unaltered between samples, and most peptides that were identified had a charge state of 2 (Figure S2B, Supporting Information). We could clearly identify the consensus anchor residues at positions 2 and 9 as expected for HLA class I‐presented peptides (Figure S2C, Supporting Information).

A large proportion of normal‐tissue‐presented peptides were also detected in matched tumor tissue, with ratios ranging from 49.4 to 71.5% (Figure 1D) while the overlap of identified sequences in technical replicate analysis of tumor or normal tissue, respectively, was 79 ± 4% on average. This demonstrated that, despite their malignant nature, tumor tissues retained certain characteristics of the tissues they derived from. In addition, there were large numbers of peptides detected only in tumor material compared to normal tissue in all patients. These results suggest that there are significant aberrant changes in the antigen presentation machinery in tumor cells, and such deviations from normal tissues could be exploited by the immune system to identify malignant cells.

Good quantitative reproducibility was observed between technical replicate analyses of normal and tumor samples in all patients (Figure S3A, Supporting information). However, poor quantitative correlation was observed in peptide abundances between normal and tumor tissues in each of the patients, respectively, with Spearman correlation values ranging between *r* = 0.07 and 0.68 (Figure S3B, Supporting information). This was expected due to the polygenic and polymorphic nature of HLA and the complex and highly diverse peptide pools that were obtained from different individuals.

### 3.2 HLA‐A2‐Positive Patients Share Tumor Peptide Antigens

**Figure 2:**
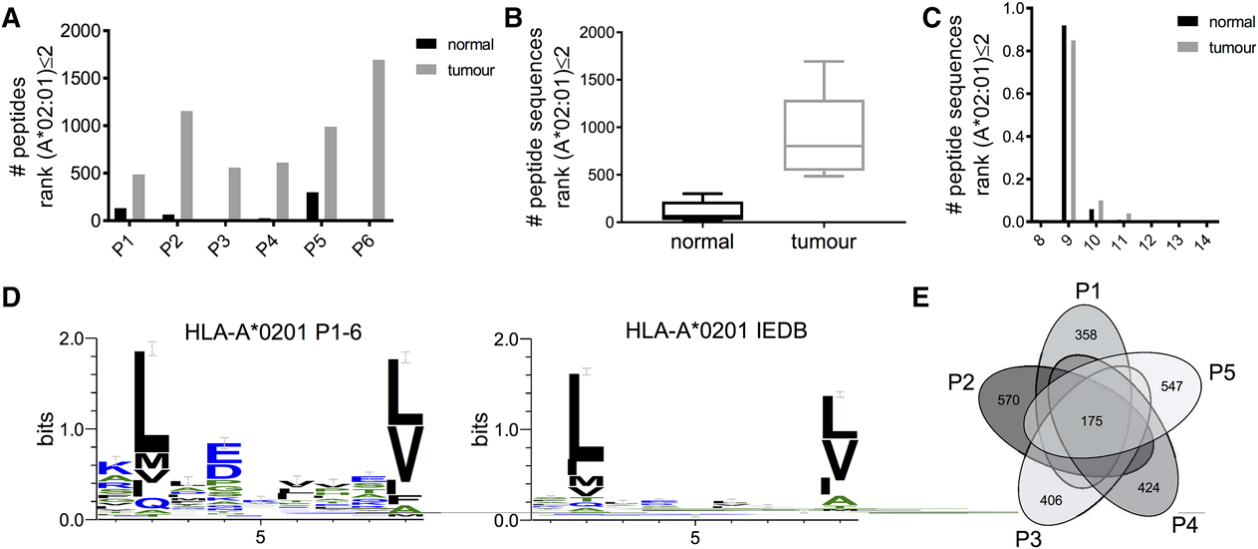
Characteristics of peptides predicted to bind HLA‐A*0201 by NetMHCpan. A) Numbers of peptides with NetMHCpan rank score for HLA‐A*0201 binding ≤2 from each patient sample. B) Average numbers and C) length distribution of all such predicted HLA‐A*0201 binders. D) Motif analysis of predicted HLA‐A*0201 binders in comparison with the known IEDB motif. E) Overlap of HLA‐A*0201‐peptides identified in all five patients.

Next we used Gibbs clustering and binding prediction with NetMHCpan to define peptides that likely originated from HLA‐A*0201 in all six patients. Clustering peptides for sequence similarity resulted in the separation of the known binding motif for HLA‐A*0201 for all six patients (Figure S4, Supporting information). A range of 581–1696 peptide sequences (13–27%) in the six patients and overall 2601 peptides (20%) of all identified peptide sequences with a length between 8 and 14 aa had attributes of HLA‐A*0201 binding with a NetMHCpan rank score equal or smaller than 2 (Figure 2A). In line with the overall peptide yield, most of such peptides also originated from tumor tissues (Figure 2B). The length distribution of HLA‐A*0201‐binding peptides showed 9‐mers were more prominently represented (Figure 2C) than that in the original immunopeptidomes (Figure 1C). Peptide motifs of the 9‐mer sequences concurred with the known binding motif of HLA‐A*0201 constructed by HLA‐A*0201‐peptide sequences deposited in the Immune Epitope Database (IEDB, Figure 2D).

A total of 175 predicted HLA‐A*0201‐peptide sequences were shared between all patients (Figure 2E), and quantitative analysis revealed that 130 of them were presented at higher levels in tumor tissue in comparison to normal tissue, and 32 of these sequences were not identified in normal tissue (Table S2, Supporting Information). Out of these 130 tumor‐associated HLA‐A*0201‐peptides, 129 were also detected in the tumor sample of Patient 6 (which had no matched adjacent normal tissue for analysis). These tumor‐associated peptides were derived from 123 distinct source proteins, of which eight proteins were represented by two peptides (Table 2). A considerable number of the 123 proteins, including some of those represented by multiple peptides, have been shown to be involved in cancer biology (Table S3, Supporting Information).

**Table 2:** Proteins represented by two common HLA‐A*0201‐peptides

UniProt acc.	Protein description	HLA‐peptides	A*0201 Rank [%]
O00560	Syntenin‐1 (SDCBP)	LMDHTIPEV SLMDHTIPEV	0.039 0.012
Q15011	Homocysteine‐responsive endoplasmic reticulum‐resident ubiquitin‐like domain member 1 protein (HERPUD1)	KMPEINAKV LLPEGPPAI	0.074 0.063
Q99613	Eukaryotic translation initiation factor 3 subunit C (eIF3c)	SLYDYNPNL SLDQPTQTV	0.005 0.079
Q9Y678	Coatomer subunit gamma‐1 (COPG1)	ALVSSLHLL AIVDKVPSV	0.124 0.013
P62906	60S ribosomal protein L10a (RPL10A)	NMVAKVDEV TLYEAVREV	0.352 0.017
O43491	Band 4.1‐like protein 2 (EPB41L2)	SLDGAPIGV SMYGVDLHHA	0.033 0.385
Q8IYM9	E3 ubiquitin‐protein ligase (TRIM22)	HLANIVERV NIVERVKEV	0.043 1.448
O14791	Apolipoprotein L1 (APOL1)	ALADGVQKV ALADGVQ^d^KV	0.010 0.010

Modified amino acids are depicted in bold, peptide modifications are abbreviated as follows: d, deamidation (+0.98 Da).

### 3.3 Identification of Protein Antigens with Tumor‐Enrichment Presentation

Following the determination of tumor‐associated HLA‐A*0201‐peptide antigens, we further asked the question that which proteins had the highest antigen presentation coverage in this cohort of TNBC tumors. We first selected proteins that were indicated to have tumor‐enriched presentation, that is, those for which all detected HLA‐peptides were presented at higher abundance in tumors compared to normal tissues in any of the five patients. A total of 171 proteins were identified, of which 112 had been previously identified through the above‐mentioned common HLA‐A*0201 peptide approach. Most of the proteins were represented by a single peptide in this dataset, but 23 proteins had two or more tumor‐associated peptides.

A number of proteins in this list had relatively large molecular weights, raising the question of whether the protein length could potentially correlate with the number of detected peptides, which may distort the above selection process. We therefore looked at the relationship of protein length and the number of detected peptides across 4812 proteins for which quantitative data was available. No linear relationship between the protein length and the number of peptides could be established (Figure S5, Supporting Information), suggesting that the enriched presentation of larger proteins was not due to their size, but rather because these proteins were intrinsically involved in or related to tumor biological processes, and were therefore preferentially processed and presented by tumor cells.

In order to prioritize the most relevant antigens, we introduced an average tumor‐associated cohort coverage (aTaCC) value for each protein that would allow us to shortlist protein antigens with the highest coverage of tumor‐associated antigen presentation across all patients. For this, we determined the protein coverage for each antigen by identifying all HLA‐presented peptides derived from the antigen in each patient, and then calculating the average coverage for each antigen across the current cohort.

aTaCC =1P∗M∑i=1P∑j=1Nlij with N≥2

where *P* is the number of patients in the cohort, *M* is the length of the protein in number of amino acids, *N* is the number of peptides derived from a particular protein presented by each patient, and *l* is the number of nonoverlapping amino acids which are covered by HLA‐presented peptide *j* in patient *i*.

The resulting aTaCC value reflects the average percentage of sequence coverage achieved by a particular protein across the analyzed cohort. Values ranged from 0.7 to 16.3% for the 23 proteins with a minimum of two tumor‐enriched peptides presented in each patient. Cofilin‐1 (CFL‐1), interleukin‐32 (IL‐32), proliferating cell nuclear antigen (PCNA), syntenin‐1 (SDCBP), and ribophorin‐II (RPN‐2) were the antigens identified as having the highest cohort presentation coverage, all with aTaCC values above 7% (Table 3; Figure S6, Supporting Information). All five proteins have significant involvement in cancer biology, in particular, syntenin‐1, which was also identified through the previous common peptide approach. We propose that the proteins with high aTaCC values could be assessed for their potential as targets for developing novel immunotherapeutics for breast cancer.

**Table 3:** Proteins with the highest tumor‐enriched cohort coverage (aTaCC ≥ 7%)

Uniprot acc.	Protein description	Protein length [aa]	Peptides/patient	aTaCC [%]	Peptides identified
P23528	Cofilin‐1 (CFL‐1)	166	3.6	16.3	IILEEGKEILV | ILEEGKEILV | SAVISLEGKPL | DGVIKVFNDMKVRKSSTPE | GVIKVFNDMKVRKSSTPE | MIYASSKDAIK | VIKVFNDMKVRKSSTPE | **A** ^a^SGVAVSD | GSAVISLEGKPL
P24001	Interleukin‐32 (IL‐32)	234	4.0	11.4	KVMRWFQAM | KVVALVHAV | DMKKLKAR**M** ^o^ | GVLAWVKEK | SQHQAIERF | VKEKVVAL | WVKEKVVAL | AELEDDFKEGY | APRGDKEEL | LEDDFKEGY | V**Q** ^d^ALWKQF | VQALWKQF | YYEEQHPEL
P12004	Proliferating cell nuclear antigen (PCNA)	261	3.6	10.6	KLMDLDVEQL | SMSADVPLV | ILKKVLEAL | LM**D** ^o^LDVEQL | RLVQGSILKK | VVKMPSGEF | KL**M** ^o^DLDVEQL | TPLSSTVTL | S**M** ^o^SADVPLV
O00560	Syntenin‐1 (SDCBP)	298	6.2	8.8	LMDHTIPEV | RPFERTITM | RPFERTIT**M** ^o^ | SLMDHTIPEV | KVIQAQTAF | RLYPELSQY | L**M** ^o^DHTIPEV | SL**M** ^o^DHTIPEV | RPFERTIT**M** ^s^ | SLMDH**T** ^h^IPEV | AHKVLKQAF | KVIQA**Q** ^d^TAF
P04844	Ribophorin‐2 (RPN‐2)	631	5.4	7.1	NRMLAQQAV | NRYHVPVVV | NRYHVPVVVV | SIAPKTTRVTY | TPHQTFVRL | ALSALTARL | ATVLQKTSF | FIADSHQNF | RLDELGGVYL | RMLAQQAVK | YAAQASQAL | IYHAVAAL | RYIANTVEL | RVTYPAKAK | SEDSSVTQIY | SVASAAAVLSH | VEVEGDNRY | ATVL**Q** ^d^KTSF | NRYIANTV

Peptides presented by all six patients are underlined. Modified amino acids are depicted in bold, peptide modifications are abbreviated as follows: a, acetylation (42.01); o, oxidation (+15.99 Da); d, deamidation (+0.98 Da); s, sulphone (+31.99 Da); h, dehydration (−18.01).

## 4 Discussion

We analyzed the immunopeptidome landscape of six HLA‐A2‐positive patients in six tumor and five adjacent normal tissue biopsies and identified a total of 19 675 distinct peptide sequences across these 11 samples. We observed higher numbers of peptides in tumor tissue as compared to normal tissue biopsies, which could be due to the expected lower density of cells in normal breast tissue that mainly consists of fatty and fibrous connective tissue. In contrast, the tumor tissue will contain a very high density of cells that will lead to overall higher levels of peptide detection.

A significant number (≈20%) of peptides identified from the tumor and normal tissue samples were predicted to be derived from HLA‐A*0201, of which 130 presented at higher abundance in tumor samples than in normal tissues. A number of source proteins were represented by multiple peptides. This prompted us to look at tumor‐associated antigen presentation coverage for individual protein antigens across all identified HLA‐peptides in all patients. High aTaCC values for cofilin‐1, IL‐32, PCNA, syntenin‐1, and ribophorin‐2 suggested that these proteins were best represented with highest sequence coverage in the tumor immunopeptidomes in this cohort. These proteins have also all been previously identified to play important roles in the context of cancer.

Cofilin‐1 is a member of the actin‐depolymerizing factor (ADF)/cofilin superfamily named after its ability to form cofilaments with actin. Its main function is to assemble or dissemble actin filaments which is central to actin dynamics. Cofilin‐1 is found to be overexpressed in various cancers and is proposed to be key to cancer invasion and metastasis, in particular, in breast cancer, by promoting cell migration and chemotaxis through actin remodeling. Inhibiting the activity of the cofilin pathway is an approach that is being investigated as potential antimetastatic therapy.^23^

IL‐32 is a novel pro‐inflammatory cytokine that promotes the production of various cytokines, such as tumor necrosis factor α (TNF‐α) and IL‐8. More recently, it has been shown to play certain roles in various cancer types.^24^ The precise mechanism of such involvement is still unknown, but IL‐32‐associated persistent inflammation, and IL‐32‐induced NF‐κB activation are possible underlying mechanisms that are being actively investigated.

Syntenin‐1 is one of the most significant tumor‐specific antigens identified in this study. It is an adaptor protein participating in cell migration, proliferation, and cell cycle regulation, and was found to be involved in the development of multiple cancer types. It is highly expressed in breast cancer cell lines and promotes cell migration in metastatic human breast cancer cells,^25^ and is associated with breast cancer progression.^26^ Therefore, it is a promising candidate target for breast cancer patients.

PCNA was originally described as an autoantigen in systemic lupus erythematosus patients.^27^ PCNA is involved in the catalysis of DNA replication and also functions in chromatin remodeling, DNA repair chromatid‐cohesion, and cell cycle control.^28^ PCNA is preferentially expressed in proliferating cancer cells, and is suggested to be a prognostic marker and potential therapeutic target for various types of cancer including breast cancer.^29^ Small‐molecule and peptide drugs targeting PCNA are in development to inhibit cancer cell growth and sensitize cells for chemotherapy and radiotherapy.

Ribophorin‐II is a subunit of oligosaccharyltransferase located on the membrane of ribosomes. RPN‐2 is highly expressed in breast cancer cells, in particular, in TNBC cancers, and correlates with metastasis and clinical tumor aggressiveness.^30^ The expression of the gene encoding RPN‐2 confers docetaxel resistance in breast cancer and RPN‐2 knockdown induces tumor cell apoptosis and sensitizes tumor cells to chemotherapy.^31^ Ribophorin‐II has also been shown to be responsible for the stabilization of mutant p53, the latter of which has 60–80% prevalence in TNBC cancers, and therefore constitutes a key driver of tumor development.^32^

Cancer associated aberrant antigen presentation can be caused by multiple mechanisms ranging from dysregulation of gene expression, protein translation, and post‐translational modification, to irregular proteasomal digestion, peptide translocation, and MHC turnover. Therefore, it is difficult to predict which peptide will be preferentially or specifically presented by tumor cells and not by normal tissues based on any one of these parameters such as protein expression level or cDNA level. After thorough optimization leading to the identification of similar peptide yields, the presented methodology has the potential to circumvent some of the difficulties experienced by genomics‐based antigen prioritization. Further validation will be required to confirm the tumor‐associated presentation of these proteins in a broad context, and to identify peptide antigens that process the best tumor‐specific presentation hence could serve as future targets for the development of targeted immunotherapy.

Our study highlights the feasibility of identifying potential targets for the development of immunotherapy from small cohort immunopeptidomics studies based on a common HLA allele. Further validation of the tumor association of such protein and peptide antigens could support wider application of such an approach in the target discovery for cancer immunotherapy.

## Conflict of Interest

The authors declare no conflict of interest.

## Supporting information

Supporting Information

Supporting Information

Supporting Information

Supporting Information

Supporting Information
