# Supplementary material for: Immunopeptidomic Profiling of HLA‐A2‐Positive Triple Negative Breast Cancer Identifies Potential Immunotherapy Target Antigens
Source: Proteomics. 2018 Jun 27;18(12):1700465. doi: 10.1002/pmic.201700465 (PMC6032843; doi:10.1002/pmic.201700465)

PROTEOMICS

Supporting Information
for Proteomics

DOI 10.1002/pmic.201700465

Nicola Ternette, Marloes J. M. Olde Nordkamp, Julius Müller, Amanda P. Anderson, Annalisa Nicastri, Adrian V. S. Hill, Benedikt M. Kessler and Demin Li

Immunopeptidomic Profiling of HLA-A2-Positive Triple Negative Breast Cancer Identifies Potential Immunotherapy Target Antigens

© 2018 The Authors. *Proteomics* Published by WILEY-VCH Verlag GmbH & Co. KGaA, Weinheim

Supporting Information Figure S1

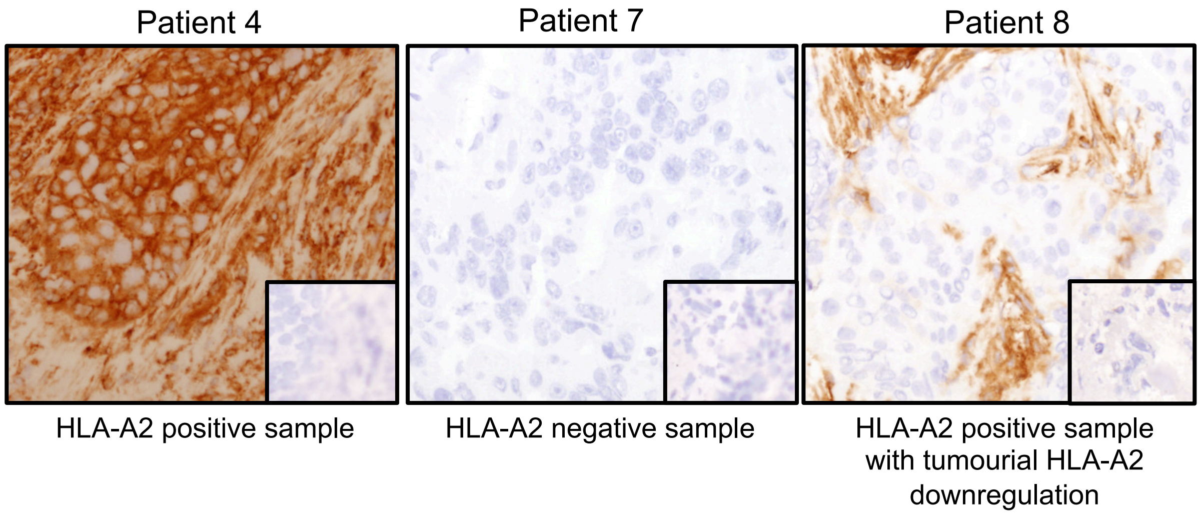

Supporting Information Figure S2

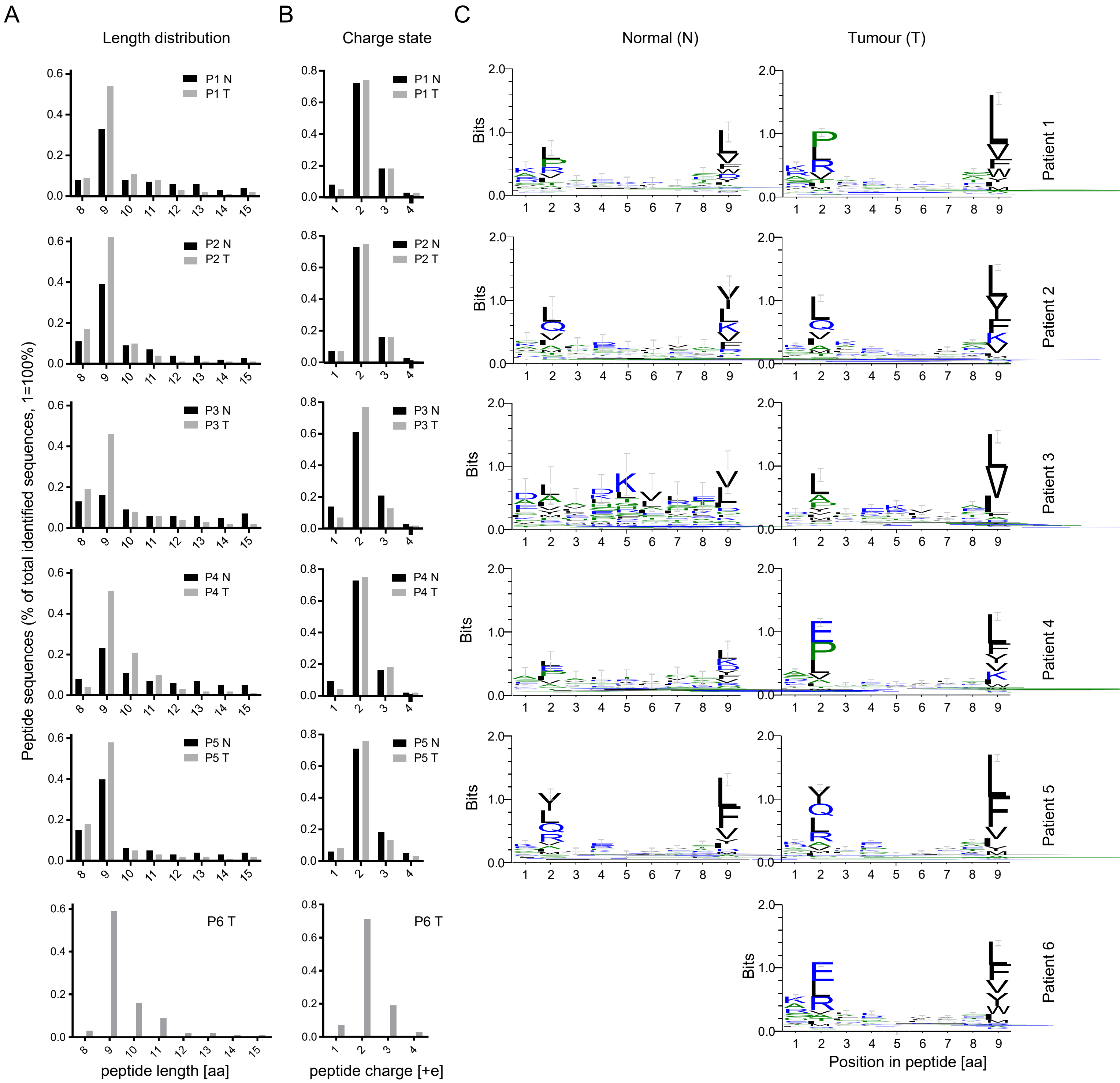

Supporting Information Figure S3

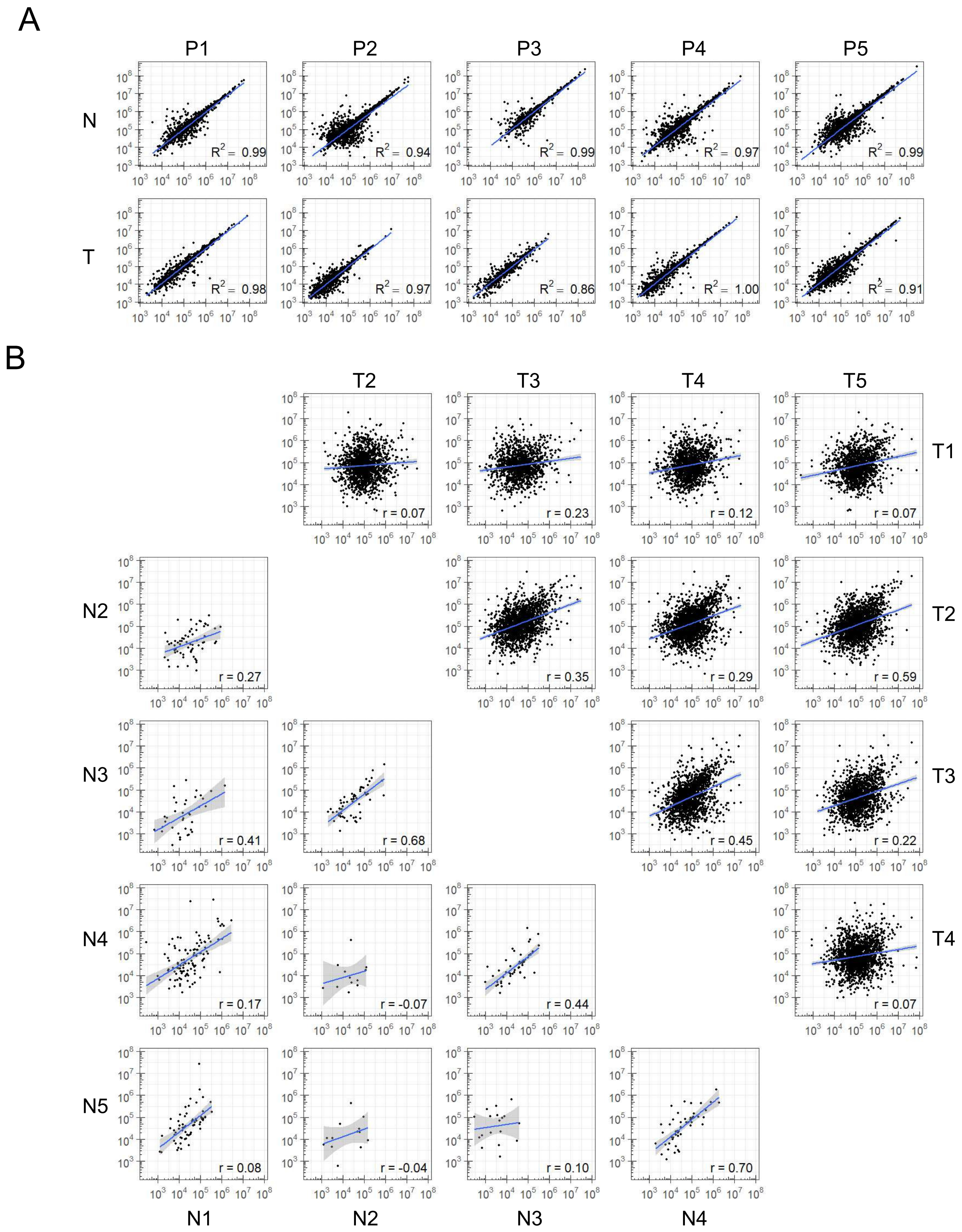

Supporting Information Figure S4

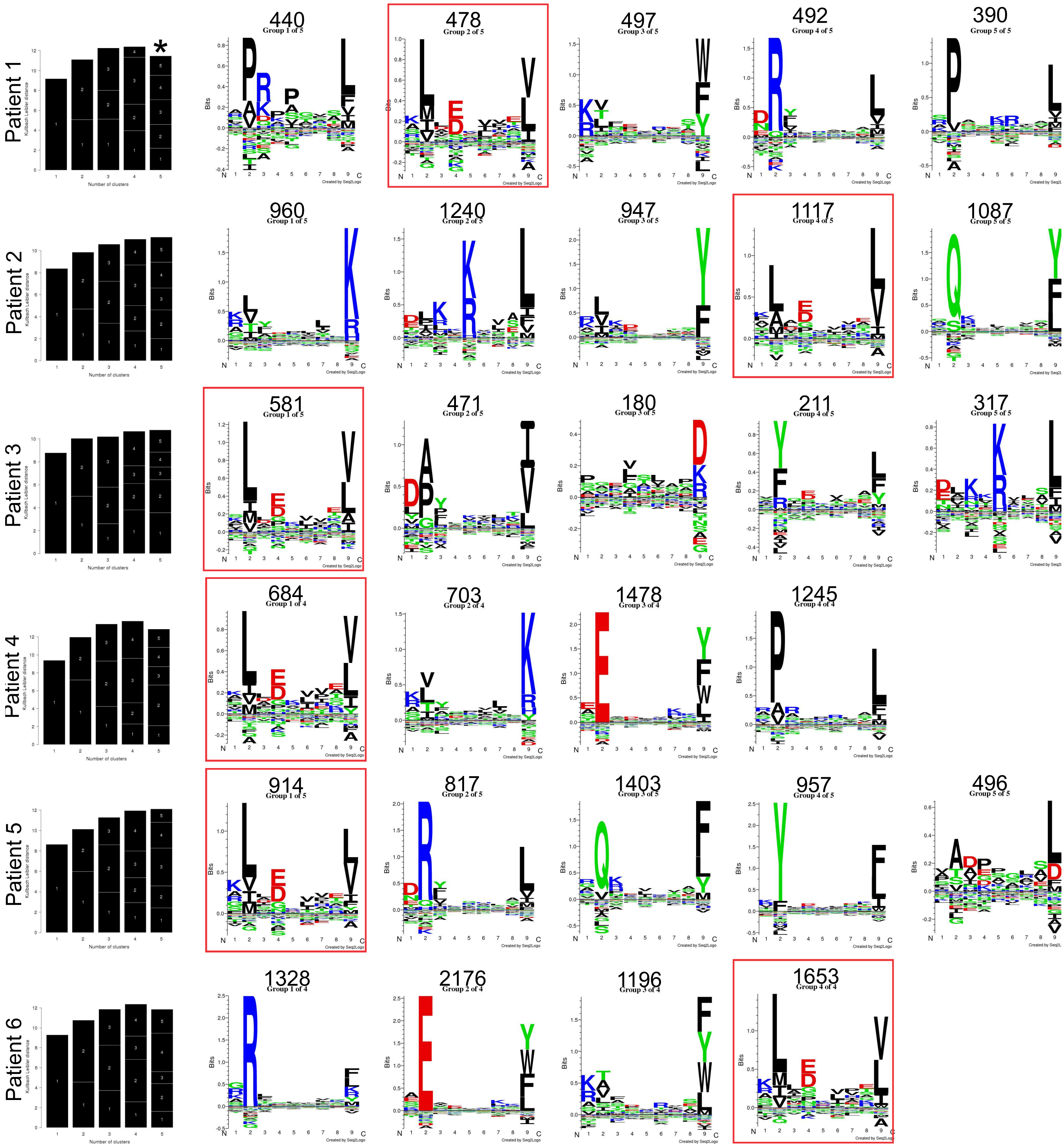

Supporting Information Figure S5

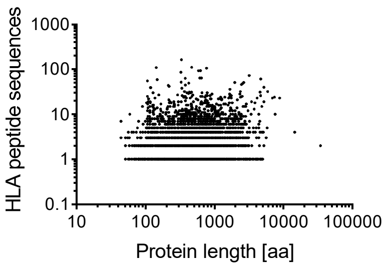

Supporting Information Figure S6

A

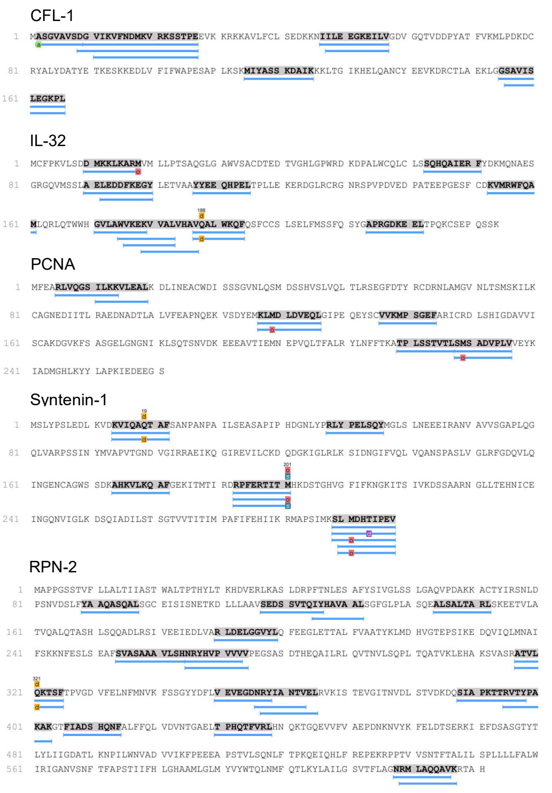

B

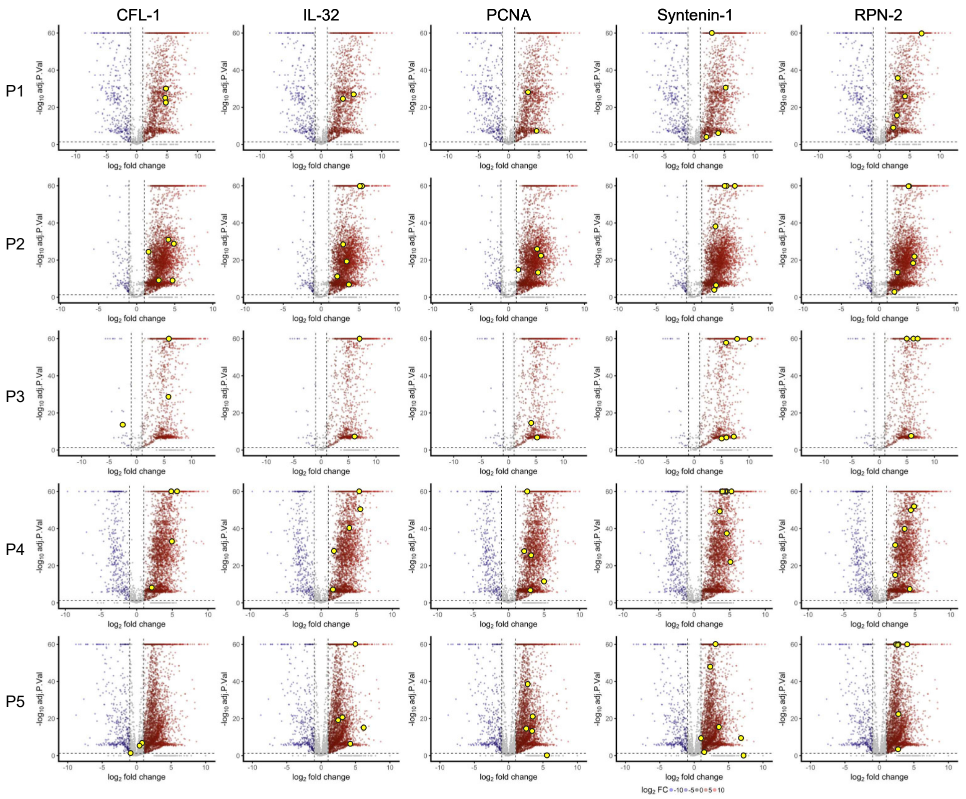

Supplement: Supplementary file 1 — Supporting Information [file PMIC-18-na-s001.pdf]
