# Supplementary material for: Immunopeptidomic Profiling of HLA‐A2‐Positive Triple Negative Breast Cancer Identifies Potential Immunotherapy Target Antigens
Source: Proteomics. 2018 Jun 27;18(12):1700465. doi: 10.1002/pmic.201700465 (PMC6032843; doi:10.1002/pmic.201700465)
Supplement: Supplementary file 5 — Supporting Information [file PMIC-18-na-s005.docx]

**Supporting Information: methods**

**Immunohistochemistry**

Immunohisto/cytochemistry for HLA-A2 was performed on acetone-fixed frozen tissue sections according to standard protocols, using the anti-HLA-A2 mouse monoclonal antibody (clone BB7.2, IgG2b, BioRad cat # MCA2090) and an isotype-matched control antibody (BioTechne, cat # MAB004), each at 10μg/ml, diluted in PBS). Briefly, slides were incubated in a solution of hydrogen peroxide (0.3% H_2_O_2_, 0.1% NaN_3_ in PBS) for 10 minutes to block any endogenous peroxidase activity. Following washes (once in PBS then once in PBS-Tween (0.05% v/v), 3 minutes each wash), slides were incubated in primary antibody solution for 30 minutes. After washing as above, the slides were subsequently incubated in secondary antibody reagent (Dako REAL ™ EnVision™ Detection System, K5007) for 30 minutes. Labelling was visualised using the Liquid DAB+ Substrate Chromogen System (Dako, K3468), allowing the colour to develop for 10 minutes. After washing as above, cells were counter-stained with Gill 3 Hematoxylin (Thermo Scientific, 6765009) and coverslips were mounted using Aquatex (VWR 1.08562.0050). All steps were performed at room temperature in a humidified chamber.

Slides were examined using an Olympus BX51 microscope with an Olympus DP70 digital camera attachment. Images were acquired using Olympus DP70 Controller software and background whiteness adjusted using Adobe Photoshop.

**Supporting Information**

**Figure S1. Selection of patient samples based on HLA-A2 expression.** TNBC tumour frozen sections were stained with the anti-HLA-A2 antibody BB7.2. Tumour samples showing positive HLA-A2 expression were chosen for MS analysis (e.g. Patient 4). Samples showing no HLA-A2 expression (e.g. Patient 7) or downregulated expression in tumour cells (e.g. Patient 8) were excluded. Insets, isotype control antibody staining.

**Figure S2. HLA-associated immunopeptidome analysis of human breast tissue.** (A) The total number of identified peptides for each patient and (B) for normal and tumour tissue from all patients is depicted showing a greater number of peptides in the tumour tissue compared with normal tissue. (C) Tumour tissues showed a greater proportion of 8-12-mer sequences than normal tissues. (D) Graphs show the peptide precursor length distribution and (E) charge state distribution as measured by MS for each patient in normal (N) and tumour (T) tissue. (F) Motifs of common amino acids are depicted for each sample for all 9-mer peptide sequences.

**Figure S3. Correlation analysis of technical replicates and tumour and normal tisuues in each of the patients, respectively.** (A) Abundances peptides in the two technical replicate analyses of normal (N) and tumour (T) tissue of each patient (P) and (B) between patients are shown as indicated.

**Figure S4. Gibbs Clustering reveals A*02:01 motif in all 6 patients.** Each row shows the Gibbs Clustering results for each of the six patients as indicated. The bar chart shows the Kullbach-Leibler distance for the solution with increasing numbers of clusters (1-5). The regarding peptide motifs for the optimal solution (with exception of P1) are displayed for each patient, and the numbers of peptide sequences in each cluster are indicated above the graphs. The regarding HLA-A*02:01 motif is highlighted by a red frame.

**Figure S5. Correlation analysis of source protein length and HLA-restricted peptide count.** The number of distinct peptide sequences identified in this cohort for each protein are plotted against the length of the deriving protein.

**Figure S6. Protein coverage for the most presented cancer-specific antigens.** (A) Peptide presentation for the proteins with the highest aTeCC values. Peptides identified from this study are indicated as blue bars below the protein amino acid sequence and peptide modifications were indicated in coloured boxes within the blue bars. Modifications are abbreviated as follows: a: acetylation (42.01); o: oxidation (+15.99 Da); d: deamidation (+0.98 Da); s: sulphone (+31.99 Da). (B) Volcano Plots depicting individual peptides for each patients. Peptides derived from the 5 most presented antigens are highlighted in yellow.
